# Episodic fluid venting from sedimentary basins fueled by pressurized mudstones

**DOI:** 10.1073/pnas.2312152121

**Published:** 2024-02-12

**Authors:** Luke M. Kearney, Richard F. Katz, Christopher W. MacMinn, Chris Kirkham, Joe Cartwright

**Affiliations:** ^a^Department of Earth Sciences, University of Oxford, Oxford OX1 3AN, United Kingdom; ^b^Department of Engineering Science, University of Oxford, Oxford OX1 3PJ, United Kingdom

**Keywords:** fluid venting, overpressure, sedimentary basins, carbon dioxide sequestration

## Abstract

Subsurface sandstone reservoirs sealed by overlying, low-permeability layers provide capacity for long-term sequestration of anthropogenic waste. Leakage can occur if reservoir pressures rise sufficiently to fracture the seal. Such pressures can be generated within the reservoir by vigorous injection of waste or, over thousands of years, by natural processes. In either case, the precise role of intercalated mudstones in the long-term evolution of reservoir pressure remains unclear; these layers have variously been viewed as seals, as pressure sinks, or as pressure sources. Here, we use the geological record of episodic fluid venting in the Levant Basin to provide striking evidence for the pressure-source hypothesis. We use a Bayesian framework to combine recently published venting data, which record critical subsurface pressures since ∼2 Ma, with a stochastic model of pressure evolution to infer a pressure-recharge rate of ∼30 MPa/Myr. To explain this large rate, we quantify and compare a range of candidate mechanisms. We find that poroelastic pressure diffusion from mudstones provides the most plausible explanation for these observations, amplifying the ∼3 MPa/Myr recharge caused primarily by tectonic compression. Since pressurized mudstones are ubiquitous in sedimentary basins, pressure diffusion from mudstones is likely to promote seal failure globally.

## Significance

Sedimentary basins can provide the capacity to sequester carbon dioxide and store hydrogen fuel. However, reliable containment requires a robust, impermeable seal. Natural fluid vents in sedimentary basins demonstrate that even highly impermeable salt seals can be breached, allowing fluids to escape. We investigate the pressure dynamics associated with fluid venting in the Levant Basin as a means to better understand the conditions leading to seal failure. We show that, contrary to what is commonly assumed, mudstone sedimentary layers can act to store pressure and feed it into reservoir layers. This helps to explain the unexpectedly high frequency of venting. Hence, it is important to measure the pressure stored in mudstones during risk assessment of sequestration and borehole drilling projects.

Sedimentary successions often include high-permeability sandstone units enveloped by thick, low-permeability mudstone units. Because the surrounding mudstones can act as barriers to fluid leakage, these sandstones are often viewed as sealed reservoirs and therefore as targets for the large-scale sequestration of waste or storage of sustainable fuels (1–3). However, fluid injection can pressurize such a reservoir to the point of triggering hydraulic fractures that breach the mudstone seal, enabling rapid depressurization by fluid venting. This mechanism of sediment depressurization has been recognized for several decades (4–6). It is generally believed that pressures below this failure threshold will dissipate by poroelastic diffusion through sealing mudstones over thousands of years (7–10). However, this slow depressurization relies on the assumption that the mudstones themselves will remain at low pressure over these long timescales, whereas a variety of natural mechanisms are known to gradually pressurize the entire sedimentary column (11). Luo and Vasseur (9) showed that overpressured mudstones can, in theory, act as a pressure source rather than as a pressure sink, re-pressurizing a sandstone reservoir after natural fluid venting. They proposed that this mechanism could fuel further episodes of venting. Kearney et al. (12) recently developed a poroelastic model of episodic venting that supports and extends this basic concept. However, the predictions of these theoretical studies are difficult to test against observational evidence due to the long timescale associated with mudstone pressure evolution.

**Fig. 1.**
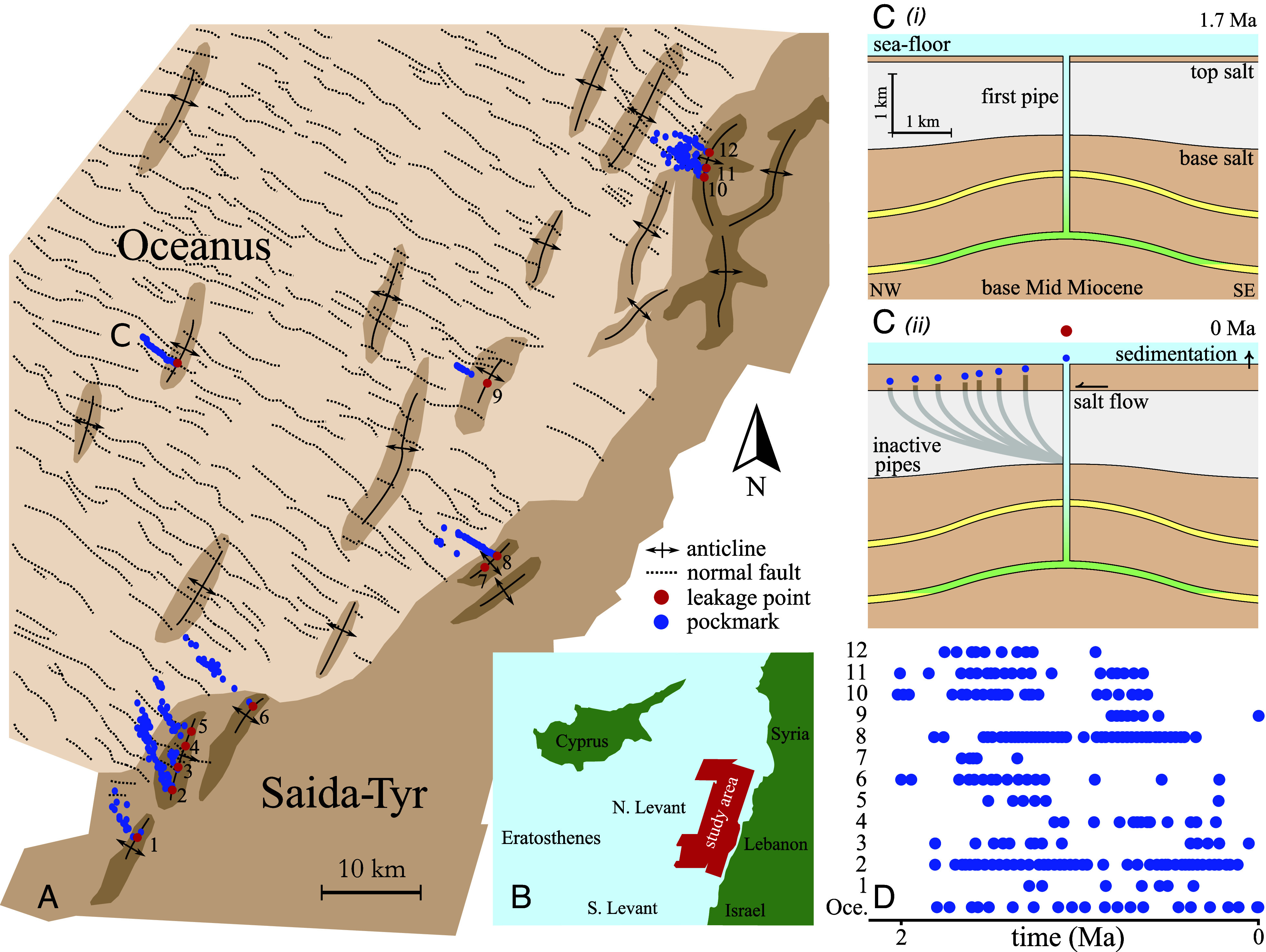
Fluid escape pipe trails in the Levant Basin. (*A*) Overview of base-salt surface, showing sub-salt anticlines and the elevated margin platform, adjacent to the normally faulted deeper basin; adapted from Oppo et al. (13), where lighter colors indicate larger depth. (*B*) Study area located on the North Levant Basin margin, offshore Lebanon. (*C*) General mechanism for fluid escape pipe trail formation, adapted from Cartwright et al. (14), with (*i*) as the formation of the initial pipe at 1.7 Ma and (*ii*) as the present-day arrangement. (*D*) Pipe trails labeled 1 to 12 and Oceanus from panel (*A*) when corrected for relative salt translation rates (13).

Here, we test the hypothesis that mudstones can act as sources of pressure, fueling fluid venting from sedimentary basins. The geological record of episodic fluid venting in the Levant Basin (Fig. 1*A*) provides a rare opportunity to elucidate the role of mudstones in the pressure evolution of sedimentary basins. These vents release overpressure in localized fluid-expulsion events that transport fluid through kilometers of low-permeability rock via cylindrical conduits known as fluid-escape pipes (14). These pipes provide a high-permeability pathway to the surface, where they terminate as pockmarks, each recording a discrete episode of venting. Field observations of relict fluid-escape pipes consistently show evidence of fracturing (15–17), suggesting that these pipes form by hydraulic fracturing (18). Hydraulic fracturing typically requires the pore pressure to exceed the local compressive stress; indeed, drilling in a region of active venting has revealed near-lithostatic pore pressures (19). Furthermore, the resulting pockmarks enable stratigraphic estimates of the time of each venting episode and thus constrain the rate of pressure recharge between episodes.

## The Levant Basin

In the North Levant Basin, located in the Eastern Mediterranean (Fig. 1*B*), more than 300 fluid-escape pipes have been documented, recording episodic fluid venting from 13 fixed locations across the region. For one of these locations, named Oceanus (Fig. 1*C*), Cartwright et al. (20) calculated that the initiation of venting via hydraulic fracturing requires ∼30 MPa of overpressure. Tectonic compression and marginal uplift have been proposed as the main overpressuring mechanisms in the region (13, 20). The Levant Basin resides within a compressive tectonic regime stemming from the collision of the African and Eurasian plates. We estimate the strain at Oceanus to be less than 10% (*SI Appendix* S1). Within the Levant Basin is a ∼3 km-thick Oligo-Miocene clastic succession consisting of turbiditic sandstones of Late Oligocene to Early Miocene age that are encased by mudstone. Many of these sandstone reservoirs host biogenic methane accumulations in NE–SW trending anticlines. The Levant pipes source methane and water from these anticline reservoirs and terminate at the seafloor as pockmarks (Fig. 1*A*). The pipes penetrate through a ∼1.5-km-thick layer of salt deposited during the Messinian Salinity Crisis (21). Recent activity of the Levant Fracture System has been uplifting the eastern margin of the basin, leading to gravity-driven, basinward salt flow since ∼2 Ma, contemporaneous with the formation of pipes in the area.

Each pipe forms vertically but the basinward viscous flow of salt advects existing pipes away from their initial positions, such that subsequent venting from the same reservoir requires the formation of a replacement pipe (Fig. 1*C*). Repetition of this process leads to the 13 observed trails of pipes in the North Levant Basin (13, 14). Thus, each pipe trail records episodic fluid venting from a single reservoir, suggesting that these reservoirs are repeatedly repressurized. From the spatial distributions of pockmarks within each pipe trail, the time of formation of each pipe can be estimated (Fig. 1*D*) using the methods of Oppo et al. (13) and Cartwright et al. (20). These approaches reveal that for each trail, pipe formation typically occurs every ∼100 kyr. Since fluid-escape pipes record critical subsurface pressures, the Levant pipe trails enable us to distinguish between theories for pressure redistribution between sedimentary layers. The timings of the pockmarks of the isolated Oceanus pipe trail are particularly well constrained as it is situated in a less tectonically active region of the basin. Oceanus is therefore less susceptible to local stress changes that might affect the recharge mechanics. We thus focus our analysis on the Oceanus trail. The remaining 12 trails are distributed along the active basin margin and are used to extend our inferences from Oceanus to a more complex system.

To test the pressure-source hypothesis, we develop a stochastic model of reservoir pressure evolution and use it to invert the Levant pipe trail data under a Bayesian framework for model parameters such as the pressure-recharge rate. Using basic physical arguments, we then estimate recharge rates for each candidate overpressure mechanism and compare with the inferred rates. In particular, Kearney et al. (12) showed that pressure diffusion from mudstones amplifies the rate of pressure recharge generated by tectonic compression. In mudstone-dominated basins like the Levant Basin, pressure-recharge rates can be amplified by a factor of ∼10. Therefore, if this hypothesis is correct, then we expect that the inferred recharge rate is a factor of ∼10 greater than that predicted for tectonic compression alone.

## Stochastic Model of Pressure Evolution

We assume that a fluid-escape pipe forms via hydraulic fracturing when the pore pressure exceeds the critical fracture pressure $p_{f}$, which is the sum of the minimum horizontal compressive stress $\sigma_{\min}$ and tensile strength $\sigma_{T}$ of the overlying mudstone (22, 23),

[1] $$\begin{array}{c} p_{f}=\sigma_{\min}+\sigma_{T}, \end{array}$$

where we take compression to be positive. Once venting begins, the pressure drops rapidly until the pathway closes, which we assume occurs when the pressure reaches $\sigma_{\min}$. Once closed, we expect fractures to self-seal via swelling and mineral precipitation (24). Roberts and Nunn (6) predict fluid venting durations of order years, which may be considered instantaneous relative to recharge times, of order 100 kyrs. Over the latter timescale, pressure will become spatially uniform within a high-permeability reservoir. We thus assume that reservoir pressure depends on time only. For multiple venting episodes to be sourced from the same reservoir, the reservoir pressure must recharge between episodes. We consider generic pressure recharge at an average rate Γ, such that the corresponding time Δt between events is:

[2] $$\begin{array}{c} \Delta t=\frac{p_{f}-\sigma_{\min}}{\Gamma }=\frac{\sigma_{T}}{\Gamma }. \end{array}$$

Fractures exploit pre-existing rock weaknesses that change over geologic time, such that $\sigma_{T}$ will vary between events. We model this variability by asserting that $\sigma_{T}$ is a normally distributed random variable with mean $\bar{\sigma_{T}}$ and SD $s_{T}$. Eq. **2** then implies that $\Delta t∼N(\bar{\sigma_{T}}/\Gamma ,s_{T}/\Gamma )$. Thus, the mean and SD of inter-event times of a trail of pockmarks can be used to infer the underlying recharge rate.

As this is a limited dataset that has been produced by an inherently stochastic process, Bayesian inference is used to invert the pipe trail data for the full probability distribution of each parameter and quantify their uncertainty. Our prior estimates of each parameter ($\Gamma ,\bar{\sigma_{T}},s_{T}$) are updated by evaluating the data with a likelihood function to recover the posterior probability distributions of each parameter. The likelihood function provides a statistical measure of model–data agreement by calculating the probability of observing the data given a set of model parameters. The simplicity of our physical model enables the likelihood function to be expressed analytically (*SI Appendix* S2). We apply a conservative Gaussian prior for $\bar{\sigma_{T}}\;∼\;N\left(2.0,1.0\right)$ MPa, since mudstone tensile strengths are typically a few MPa (25, 26); in particular, Roberts and Nunn (6) predict a pressure drop of ∼2 MPa from venting. The posterior distributions of each parameter are sampled using the Metropolis–Hastings algorithm (27).

## Oceanus Pockmark Trail

**Fig. 2.**
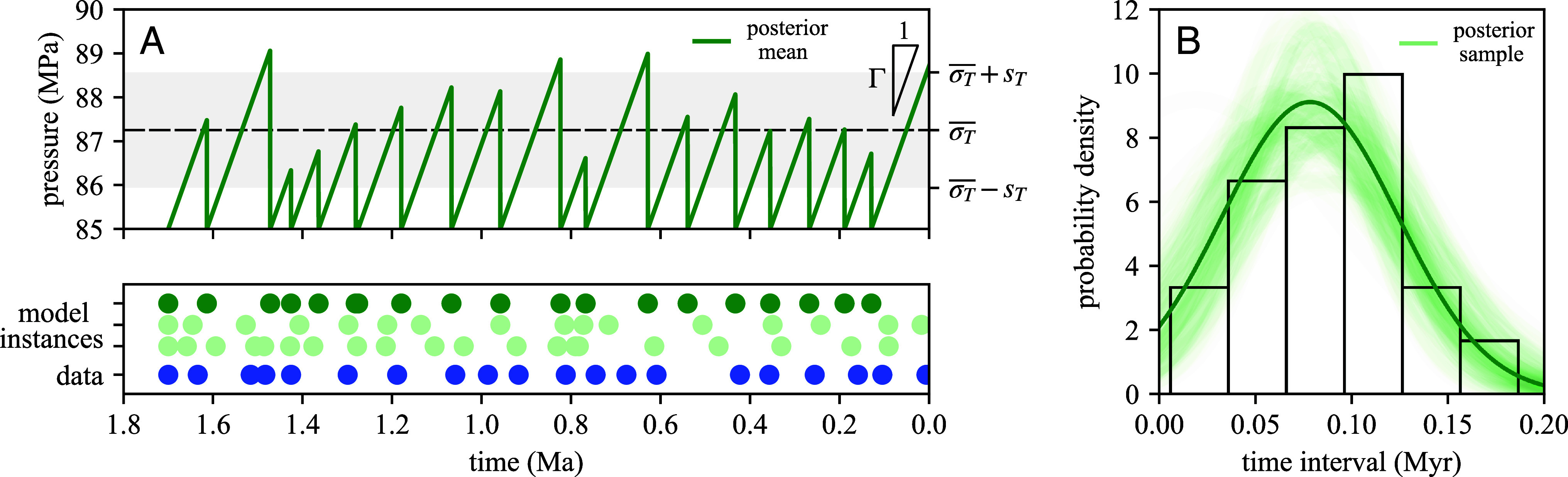
Results of Bayesian inference applied to the Oceanus pockmark trail. (*A*) *Lower* panel: Time-transformed pockmark data (blue) and stochastic model instances of venting history using inferred posterior mean (dark green) and sample parameters (translucent green). *Upper* panel: Stochastic instance of linearized pressure evolution using inferred posterior mean parameters (dark green), with pressures in excess of the minimum compressive stress corresponding to the mean tensile strength (dashed line) and corresponding to tensile strength values within one SD of the mean (gray-shaded area). (*B*) Posterior mean (dark green) and sample (translucent green) time interval distributions compared with Oceanus data.

From the posterior distributions inferred for the isolated Oceanus trail (*SI Appendix* S4), we use the mean posterior parameter values as input for our stochastic model to simulate an instance of linearized pressure evolution (Fig. 2*A*). The qualitative similarity between the pockmark data and the model output is apparent (Fig. 2*A*, *Lower* panel). For statistical comparison, we use samples from the posterior parameter distributions to calculate a range of posterior time interval distributions (Fig. 2*B*) that agree well with the data; variations between samples indicate the level of uncertainty in the inference. We note that as we have inferred the time-averaged recharge rate, this linearized pressure evolution resembles the sawtooth behavior that is predicted for recharge from tectonic compression only (12, 20). However, our statistical model makes no physical assumptions regarding the mechanism or dynamics of pressure recharge between venting episodes.

## Levant Margin Pockmark Trails

To the east of Oceanus are 12 other trails distributed along the basin margin (13). Some of these trails originate from the same anticline, separated only by ∼1 km, and thus may be in hydraulic communication. To account for this possibility, we introduce pressure coupling as a feature of the model. For a coupled system of pipes, after any one pipe vents, the pressures of all pipes coupled to it reset to $\sigma_{\min}$ and a new $\sigma_{T}$ is sampled for each. Therefore, the pipe that vents pressure temporarily inhibits any coupled pipes from venting. Consequently, the pipes in a coupled system form complementary pockmark series (*SI Appendix* S5). If a group of pipes are instead uncoupled, each pipe behaves independently. This contrast between independent and complementary venting behavior is a qualitative diagnostic for pressure coupling.

To evaluate whether a pair of adjacent trails are coupled, we calculate the Bayes factor of the coupled model $M_{c}$ and uncoupled model $M_{u}$ (*SI Appendix* S2). The Bayes factor $B_{\mathrm{cu}}$ of two models $M_{c}$ and $M_{u}$ is given by the ratio of probabilities of observing the data t given each model, i.e.,

[3] $$\begin{array}{c} B_{\mathrm{cu}}=\frac{P(t\;|\;M_{c})}{P(t\;|\;M_{u})}. \end{array}$$

For example, if $B_{\mathrm{cu}}>1$, then $M_{c}$ is preferred over $M_{u}$. Kass and Raftery (28) state that Bayes factors in the range 10 to 100 are “strong” and above 100 are “decisive.” We use this interpretation to assess the couplings of the pipe trails.

**Fig. 3.**
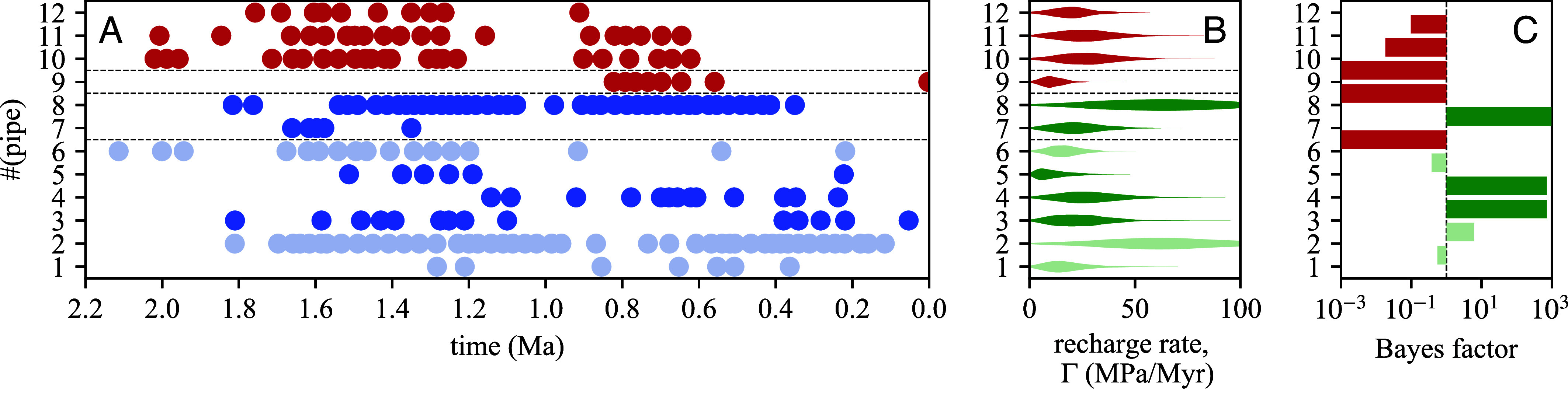
Results of Bayesian inference applied to Levant margin data. (*A*) Time-transformed data from Oppo et al. (13). Dashed lines divide pipe clusters that are separated by more than 10 km. (*B*) Violin plot of posterior recharge rate distributions for each pipe trail. (*C*) Bayes factors of pairwise pipe analysis, where a positive value implies the coupled model is more likely.

For the Levant margin pipe data (Fig. 3*A*), we infer similar recharge rates to those inferred for Oceanus, although mean recharge rates range up to 66 MPa/Myr for pipe trail 8 (Fig. 3*B*). Fig. 3*C* shows Bayes factors of pairwise analysis of adjacent trails. Triple-wise analysis leads to the same conclusions but has been omitted to simplify the interpretation (*SI Appendix* S6). The model identifies all adjacent pipes that are greater than 10 km apart as decisively uncoupled. Furthermore, the inverted model indicates hydraulic connectivity between pipes 3, 4, and 5, each located along the same anticline, as well as trails 7 and 8 (Fig. 3*C*).

The inferences for pressure coupling are in agreement with the qualitative diagnostic behavior. For example, the complementary venting behavior of trails 3, 4, and 5 is visually evident. Conversely, trails 10, 11, and 12 are statistically inferred to be uncoupled and exhibit independent venting behavior. Bayes factors with magnitudes below 10 exist for trail pairs {1, 2}, {2, 3}, and {5, 6}, indicating a lack of preference for either coupling or not. We attribute this neutrality to features in the data that obscure the underlying recharge mechanics. These features are likely due to local stress variations caused by, for example, faulting. Nonetheless, since the majority of results do have strong preferences to one model or another, we assert that the physical model captures the main pressure behavior, both spatially and temporally. This result lends support to our statistical inferences of pressure-recharge rates.

## Comparison of Candidate Overpressure Mechanisms

**Fig. 4.**
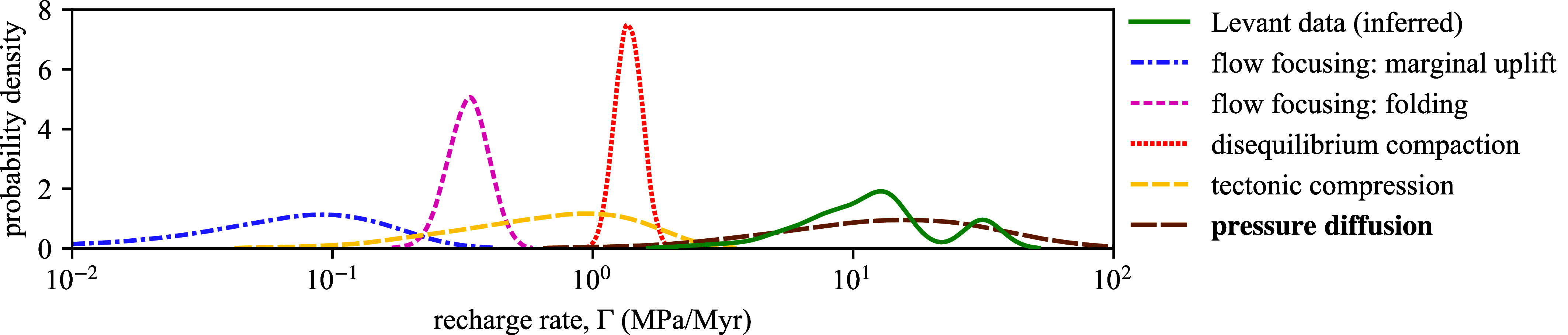
Comparison of pressure-recharge rates inferred from Levant pipe trail data with estimated recharge rates from candidate mechanisms.

The venting observations could plausibly be explained by various overpressure mechanisms that have been previously proposed. We next show that these mechanisms are inconsistent with our inferred recharge rates. Tectonic compression has been proposed as a major contributor to overpressure in the region (20). Previous numerical modeling of tectonic compression indicates that overpressures of 11 to 14 MPa in total can be generated from 10% strain (29). At Oceanus, the strain accumulated since at least the Messinian Salinity Crisis, 5 to 6 Ma, is less than 10%. This implies a maximum recharge rate of ∼3 MPa/Myr from tectonic compression, which is insufficient to reproduce the observations (Fig. 4). However, Kearney et al. (12) showed that pressure diffusion from mudstones amplifies the tectonic pressure-recharge rate in adjacent sandstones by a factor of (1+ν/γ). The factor is termed the venting frequency multiplier, and ν/γ is a ratio of dimensionless numbers that quantifies the relative effects of diffusion and compression. The dimensionless quantity *γ* measures the tectonic pressure-recharge rate of the sandstone relative to that of the mudstone; *ν* is hydraulic capacitance of the mudstone relative to that of the sandstone. The hydraulic capacitance of a layer is the product of compressibility and thickness. Typically, ν/γ≫1 in basins composed primarily of mudstone (12), like the North Levant Basin. Due to the wide range of uncertainty in mudstone permeabilities (10, 30), it might be expected that the uncertainty in the recharge rate from mudstone pressure diffusion would span many orders of magnitude. However, the venting frequency multiplier is independent of the mudstone permeability (12). This result enables us to calculate the recharge rate from the combined effect of diffusion and compression using prior distributions of each constituent parameter, giving a probability distribution that largely overlaps with inferred recharge rates (Fig. 4).

Other candidate mechanisms predict much lower recharge rates than those inferred from the data (Fig. 4). The details of how we estimate the pressure-recharge rates from each mechanism are found in *SI Appendix* S7. Oppo et al. (13) proposed that marginal uplift generates significant overpressures at the basin margin by driving lateral fluid migration from the highly overpressured deep basin. If pressure is transferred laterally along a connected, high-permeability sandstone unit, the venting periods would be several orders of magnitude lower than are observed. However, it is likely that there is poor lateral reservoir connectivity in the area (20) and our analysis above supports this idea, indicating that many relatively nearby pipes are likely to be hydraulically independent (Fig. 3*C*). As a result, the only pathway for lateral fluid migration is via mudstones, thus implicitly requiring pressure diffusion from mudstones for reservoir recharge. Marginal uplift may also generate overpressure by flow focusing (31), though this mechanism likely produces insufficient recharge rates (Fig. 4). Flow focusing due to fold amplification (31) merely generates overpressures at a rate less than ∼1 MPa/Myr. Furthermore, hydrocarbon generation likely cannot generate the required recharge rate since the additional head required from buoyancy is greater than ∼1 km/Myr and most thermogenic gas generation was likely complete by 5 to 6 Ma (32). We cannot rule out the possibility of weak pressure recharge from biogenic gas generation, though petroleum systems modeling of the region favors biogenic gas accumulation via lateral migration from the deep basin (33–35). However, due to poor lateral reservoir connectivity, lateral gas migration is rate-limited by pressure diffusion (as for the case of marginal uplift). While lateral transfer produces insufficient recharge rates, vertical pressure transfer from a deeper reservoir along faults or fractures has been associated with fluid venting in other regions (36–38). In the Levant Basin, however, there is no evidence to support vertical fluid migration. Moreover, vertical transfer cannot explain the observed regular periodicity of venting. Disequilibrium compaction due to the small, post-salt sediment accumulation of ∼300 m (20) creates a negligible pressurization rate of ∼1 MPa/Myr. Sea-level fluctuations may trigger venting episodes (23), though this mechanism alone provides no net pressure recharge.

The venting observations from the Levant fluid-escape pipe trails are consistent with predictions deriving from the hypothesis that pressure diffusion from mudstones fuels episodic venting in the region. Therefore, the Levant pipe trails provide strong spatiotemporal evidence supporting this hypothesis. In doing so, the pipe trails support a more general idea—that pressure diffusion from mudstones plays an important role in pressure redistribution between sedimentary layers—and provide observational evidence that was previously lacking from the theoretical literature, e.g., refs. 7–9, and 12. It is likely that tectonic compression and marginal uplift were the main mechanisms for slowly pressurizing the basin to near-lithostatic by ∼2 Ma. This pressurization initiated basin-wide fluid venting by hydraulic fracturing, sourced by high-permeability, pre-salt sandstone reservoirs. Tectonic compression continued to slowly pressurize (∼3 MPa/Myr) the entire sedimentary succession while poroelastic pressure diffusion from mudstones recharged the sandstone reservoirs back to failure at a rate of ∼30 MPa/Myr. This combination of pressure diffusion and tectonic compression, with minor contributions from hydrocarbon generation and disequilibrium compaction, led to episodic fluid venting with a typical venting period of ∼100 kyr. While this is not a universal result for pipes in any basin, pressure diffusion exists wherever the corresponding reservoir unit is encased by highly overpressured, low-permeability rocks. Furthermore, the effect of pressure diffusion is intensified in sedimentary basins composed mostly of mudstone (12), where fluid venting phenomena are commonly observed (18). In many cases, liquefied mudstone is vented in addition to basinal fluids, e.g., ref. 39. The diverse roles of mudstones in pressure-driven, focused fluid venting provides an impetus to improve our mechanistic models of such venting phenomena.

## Broader Implications

Because understanding subsurface pressure is crucial to prevent unwanted fluid leakage, these results have wider implications for risk assessment during borehole drilling and the sequestration of waste such as CO_2_. Fluid leakage resulting from reservoir pressurization by mudstones may be a risk in a broad range of geological settings, requiring only that the mudstones are overpressured relative to the reservoir. This overpressure can be retained even after several episodes of fluid venting (12) and can be generated by various means, not limited to horizontal compression. Indeed, Kearney et al. (12) show that disequilibrium compaction (i.e., vertical compression) leads to mathematically equivalent behavior. Therefore, even tectonically inactive regions like passive margins are prone to episodic venting if they are subjected to, for example, high sedimentation rates. Indeed, fluid-escape pipes are commonly observed in passive margin settings (18). Passive margins also provide the largest and likely most cost-effective large-scale CO_2_ storage resource (40). Therefore, fluid-escape pipes may pose a significant threat to offshore storage projects.

This work highlights the importance of considering pressure diffusion from mudstones when assessing reservoir overpressures. This is especially true for sequestration sites with evidence of previous fluid venting, like the Sleipner field (41, 42). While the relict fluid-escape pipes at Sleipner are unlikely to be a result of CO_2_ injection (42), they serve as an example of the risks to containment associated with fluid venting. Although the dissolution of injected CO_2_ can act to depressurize a storage reservoir (43), evidence from a natural CO_2_ reservoir suggests that the rate of depressurization from CO_2_ dissolution is ∼1 MPa/Myr (44). This is much less than the recharge rates from pressure diffusion that we infer in the Levant Basin, suggesting that CO_2_ dissolution is unlikely to prevent leakage in regions where pressure diffusion from mudstones is active. Thus, for storage projects in regions with pressurized mudstones, our results indicate that reservoir pressure monitoring over several millennia may be required to ensure containment.

## Supplementary Material

Appendix 01 (PDF)

## Data Availability

The data and code used in this work are available at https://doi.org/10.5281/zenodo.8083599 (45).

## References

[1] S. Krevor , Subsurface carbon dioxide and hydrogen storage for a sustainable energy future. Nat. Rev. Earth Environ. **4**, 1–17 (2023).

[2] N. Heinemann , Enabling large-scale hydrogen storage in porous media-the scientific challenges. Energy Environ. Sci. **14**, 853–864 (2021).

[3] P. S. Ringrose , Storage of carbon dioxide in saline aquifers: Physicochemical processes, key constraints, and scale-up potential. Annu. Rev. Chem. Biomol. Eng. **12**, 471–494 (2021). 33872518 10.1146/annurev-chembioeng-093020-091447

[4] E. Noble, Formation of ore deposits by water of compaction. Econ. Geol. **58**, 1145–1156 (1963).

[5] L. Cathles, A. Smith, Thermal constraints on the formation of Mississippi Valley-type lead-zinc deposits and their implications for episodic basin dewatering and deposit genesis. Econ. Geol. **78**, 983–1002 (1983).

[6] S. J. Roberts, J. A. Nunn, Episodic fluid expulsion from geopressured sediments. Mar. Pet. Geol. **12**, 195–204 (1995).

[7] A. Muggeridge, Y. Abacioglu, W. England, C. Smalley, Dissipation of anomalous pressures in the subsurface. J. Geophys. Res. Solid Earth **109**, B11104 (2004).

[8] A. Muggeridge, Y. Abacioglu, W. England, C. Smalley, The rate of pressure dissipation from abnormally pressured compartments. AAPG Bull. **89**, 61–80 (2005).

[9] X. Luo, G. Vasseur, Overpressure dissipation mechanisms in sedimentary sections consisting of alternating mud-sand layers. Mar. Pet. Geol. **78**, 883–894 (2016).

[10] K. W. Chang, M. A. Hesse, J. P. Nicot, Dissipation of overpressure into ambient mudrocks during geological carbon dioxide storage. Energy Procedia **37**, 4457–4464 (2013).

[11] M. J. Osborne, R. E. Swarbrick, Mechanisms for generating overpressure in sedimentary basins: A reevaluation. AAPG Bull. **81**, 1023–1041 (1997).

[12] L. M. Kearney, C. W. MacMinn, R. F. Katz, C. Kirkham, J. Cartwright, Episodic, compression-driven fluid venting in layered sedimentary basins. Proc. Royal Soc. A: Math. Phys. Eng. Sci. **479**, 20220654 (2023).

[13] D. Oppo , Leaky salt: Pipe trails record the history of cross-evaporite fluid escape in the northern Levant Basin, Eastern Mediterranean. Basin Res. **33**, 1798–1819 (2021).

[14] J. Cartwright, C. Kirkham, C. Bertoni, N. Hodgson, K. Rodriguez, Direct calibration of salt sheet kinematics during gravity-driven deformation. Geology **46**, 623–626 (2018).

[15] M. Huuse, S. J. Shoulders, D. I. Netoff, J. Cartwright, Giant sandstone pipes record basin-scale liquefaction of buried dune sands in the middle Jurassic of SE Utah. Terra Nova **17**, 80–85 (2005).

[16] K. Roberts, R. Davies, S. Stewart, Structure of exhumed mud volcano feeder complexes, Azerbaijan. Basin Res. **22**, 439–451 (2010).

[17] H. Løseth , 1000 m long gas blow-out pipes. Mar. Pet. Geol. **28**, 1047–1060 (2011).

[18] J. Cartwright, C. Santamarina, Seismic characteristics of fluid escape pipes in sedimentary basins: Implications for pipe genesis. Mar. Pet. Geol. **65**, 126–140 (2015).

[19] M. J. Reilly, P. B. Flemings, Deep pore pressures and seafloor venting in the Auger Basin, Gulf of Mexico. Basin Res. **22**, 380–397 (2010).

[20] J. Cartwright , Quantitative reconstruction of pore-pressure history in sedimentary basins using fluid escape pipes. Geology **49**, 576–580 (2021).

[21] W. B. Ryan, Decoding the Mediterranean salinity crisis. Sedimentology **56**, 95–136 (2009).

[22] N. J. Price, J. W. Cosgrove, Analysis of Geological Structures (Cambridge University Press, 1990).

[23] B. P. Scandella, C. Varadharajan, H. F. Hemond, C. Ruppel, R. Juanes, A conduit dilation model of methane venting from lake sediments. Geophys. Res. Lett. **38**, L06408 (2011).

[24] H. Bock , Self-Sealing of Fractures in Argillaceous Formations in the Context of Geological Disposal of Radioactive Waste (OECD and NEA, 2010).

[25] D. Okland, G. K. Gabrielsen, J. Gjerde, S. Koen, E. L. Williams, “The importance of extended leak-off test data for combatting lost circulation” in *SPE/ISRM Rock Mechanics Conference* (OnePetro, 2002).

[26] A. Raaen, P. Horsrud, H. Kjørholt, D. Økland, Improved routine estimation of the minimum horizontal stress component from extended leak-off tests. Int. J. Rock Mech. Min. Sci. **43**, 37–48 (2006).

[27] W. K. Hastings, Monte Carlo sampling methods using Markov chains and their applications. Biometrika **57**, 97–109 (1970).

[28] R. E. Kass, A. E. Raftery, Bayes factors. J. Am. Stat. Assoc. **90**, 773–795 (1995).

[29] J. Obradors-Prats, M. Rouainia, A. C. Aplin, A. J. Crook, Stress and pore pressure histories in complex tectonic settings predicted with coupled geomechanical-fluid flow models. Mar. Pet. Geol. **76**, 464–477 (2016).

[30] Y. Yang, A. C. Aplin, Permeability and petrophysical properties of 30 natural mudstones. J. Geophys. Res. Solid Earth **112**, B03206 (2007).

[31] P. B. Flemings, B. B. Stump, T. Finkbeiner, M. Zoback, Flow focusing in overpressured sandstones: Theory, observations, and applications. Am. J. Sci. **302**, 827–855 (2002).

[32] A. N. Al-Balushi, M. Neumaier, A. J. Fraser, C. A. Jackson, The impact of the Messinian salinity crisis on the petroleum system of the Eastern Mediterranean: A critical assessment using 2D petroleum system modelling. Pet. Geosci. **22**, 357–379 (2016).

[33] S. Bou Daher , 3D thermal history and maturity modelling of the Levant Basin and its eastern margin, offshore-onshore Lebanon. Arab. J. Geosci. **9**, 1–26 (2016).

[34] R. Ghalayini, F. Nader, S. Bou Daher, N. Hawie, W. Chbat, Petroleum systems of Lebanon: An update and review. *J. Pet. Geol* **41**, 189–214 (2018).

[35] F. H. Nader, L. Inati, R. Ghalayini, N. Hawie, S. B. Daher, Key geological characteristics of the Saida-tyr platform along the eastern margin of the Levant Basin, offshore Lebanon: Implications for hydrocarbon exploration. Oil Gas Sci. Technol.-Revue d’IFP Energies Nouvelles **73**, 50 (2018).

[36] D. Grauls, J. Baleix, Role of overpressures and in situ stresses in fault-controlled hydrocarbon migration: A case study. Mar. Pet. Geol. **11**, 734–742 (1994).

[37] M. R. Tingay, R. R. Hillis, R. E. Swarbrick, C. K. Morley, A. R. Damit, ‘Vertically transferred’ overpressures in Brunei: Evidence for a new mechanism for the formation of high-magnitude overpressure. Geology **35**, 1023–1026 (2007).

[38] L. Cathles, On the processes that produce hydrocarbon and mineral resources in sedimentary basins. Geosciences **9**, 520 (2019).

[39] J. Cartwright, C. Kirkham, D. N. Espinoza, D. James, N. Hodgson, The evolution of depletion zones beneath mud volcanoes. Mar. Pet. Geol. **55**, 106351 (2023).

[40] P. S. Ringrose, T. A. Meckel, Maturing global CO_2_ storage resources on offshore continental margins to achieve 2DS emissions reductions. Sci. Rep. **9**, 1–10 (2019). 31784589 10.1038/s41598-019-54363-z PMC6884532

[41] R. Arts , Seismic monitoring at the Sleipner underground CO_2_ storage site (North Sea). Geol. Soc. London Spec. Publ. **233**, 181–191 (2004).

[42] A. J. Cavanagh, R. S. Haszeldine, The Sleipner storage site: Capillary flow modeling of a layered CO_2_ plume requires fractured shale barriers within the Utsira Formation. Int. J. Greenhouse Gas Control **21**, 101–112 (2014).

[43] D. Akhbari, M. A. Hesse, Causes of underpressure in natural CO_2_ reservoirs and implications for geological storage. Geology **45**, 47–50 (2017).

[44] K. J. Sathaye, M. A. Hesse, M. Cassidy, D. F. Stockli, Constraints on the magnitude and rate of CO_2_ dissolution at Bravo Dome natural gas field. Proc. Natl. Acad. Sci. U.S.A. **111**, 15332–15337 (2014). 25313084 10.1073/pnas.1406076111 PMC4217453

[45] L. M. Kearney et al., Data and code for “Episodic fluid venting from sedimentary basins fueled by pressurized mudstones”. Zenodo. 10.5281/zenodo.8083599. Accessed 31 January 2024. PMC10895340 38346195

