# Supplementary material for: Episodic fluid venting from sedimentary basins fueled by pressurized mudstones
Source: Proc Natl Acad Sci U S A. 2024 Feb 12;121(8):e2312152121. doi: 10.1073/pnas.2312152121 (PMC10895340; doi:10.1073/pnas.2312152121)
Supplement: Supplementary file 1 — Appendix 01 (PDF) [file pnas.2312152121.sapp.pdf]

Episodic fluid venting from sedimentary basins fuelled by pressurised mudstones

Luke M. Kearney[1], Richard F. Katz[1], Christopher W. MacMinn[2], Chris Kirkham[1], and Joe Cartwright[1]

[1]*Department of Earth Sciences, University of Oxford, Oxford OX1 3AN, United Kingdom*
[2]*Department of Engineering Science, University of Oxford, Oxford OX1 3PJ, United Kingdom*

Supplementary material

Source code for the figures in this study is available from the Zenodo repository: doi:10.5281/zenodo.8083599.

S1. OCEANUS STRAIN

We estimate the horizontal strain at the Oceanus pipe trail from the depth-converted cross-section in Cartwright et al. (2021). We do this by measuring the arc length of one of the folded Mid-Miocene sandstone layers (Fig. S1). We assume that this layer was initially horizontal, such that the arc length of the layer measures the initial horizontal extent of this section L_0. We calculate the horizontal strain by comparing this initial horizontal extent to the present-day horizontal extent of this layer L, using

$$e_{xx} = -\frac{L - L_0}{L_0}, \tag{1}$$

where compressive strains are taken to be positive. This gives a horizontal strain of 2% at Oceanus. While the majority of strain in the region is accommodated by folding, we recognise that this calculation does not account for strain accommodated by faulting or volumetric compression. Further uncertainty in this calculation stems from potential errors in the depth migration of the seismic data. Accounting for this uncertainty conservatively, we assert that the maximum possible horizontal strain at Oceanus is 10%.

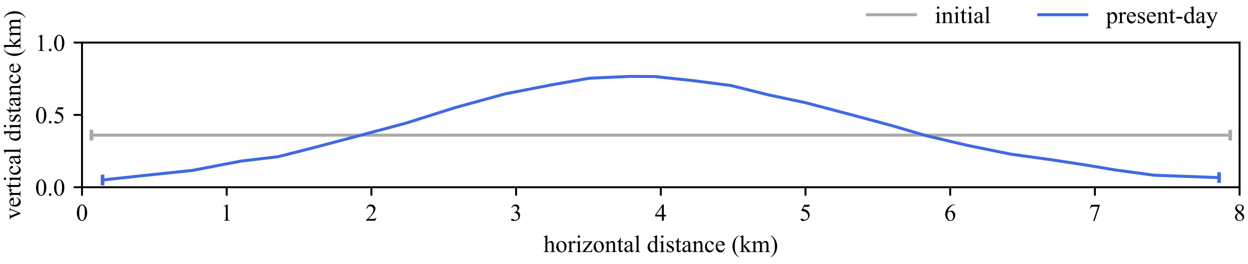

Figure S1: Comparison of the present-day geometry of the folded sandstone layer at Oceanus (blue curve) with its likely initial state (grey curve), i.e., before the onset of tectonic compression. The horizontal strain at Oceanus can be calculated by comparing the horizontal extent of the present-day fold L with the horizontal extent of its initial unfolded state L_0.

S2. BAYESIAN INFERENCE

One can use a Bayesian framework to invert for the parameters. Bayes' theorem is given by

$$\mathbb{P}(\theta \,|\, \mathbf{x}) = \frac{\mathbb{P}(\mathbf{x} \,|\, \theta)\mathbb{P}(\theta)}{\mathbb{P}(\mathbf{x})} = \frac{\mathbb{P}(\mathbf{x} \,|\, \theta)\mathbb{P}(\theta)}{\int_{\Theta} \mathbb{P}(\mathbf{x} \,|\, \theta)\mathbb{P}(\theta)\mathrm{d}\theta}, \tag{2}$$

or in words,

$$\text{posterior} = \frac{\text{likelihood} \times \text{prior}}{\text{evidence}}. \tag{3}$$

Maximum-likelihood estimation methods such as ordinary least-squares aim to maximise the likelihood, the probability that the model generates the data. This is equivalent to maximising the posterior under the assumption of a uniformly distributed prior. However, Bayesian methods place a prior distribution on the parameters and calculates the posterior distribution using the observed data. We achieve the statistical equivalent to regularisation by enforcing these prior distributions.

S2.1 Likelihood function

Given a model, the likelihood function is the joint probability of the observed data. Here, the observed data is the set of all venting times $\mathbf{t} = \{t_n\}_{n=1}^N$. The likelihood function can be decomposed in the following way:

$$f(\{t_n\}_{n=1}^N) = \prod_{n=1}^{N} f(t_n \,|\, \mathcal{H}_{t_n}), \tag{4}$$

where f is the probability density and the history $\mathcal{H}_{t_n}$ is the set of all event times until (but not including) t_n. Since the proposed model asserts that the pressure resets to $\sigma_{\min}$ after each event, the pressure 'memory' of the system extends only from the most recent event so $\mathcal{H}_{t_n} = t_{n-1}$. We can therefore write

$$f(\{t_n\}_{n=1}^N) = \prod_{n=1}^{N} f(\Delta t_n), \tag{5}$$

where $\Delta t_n = t_n - t_{n-1}$. For coupled systems we must additionally consider the mark of each pipe κ, denoting where each event originated. It can be shown that for a set of K coupled pipes,

$$f(\{t_n, \kappa_n\}_{n=1}^N) = \prod_{n=1}^{N} f_{\kappa_n}(\Delta t_n) \prod_{k=1}^{K} \Big[1 - F_k(\Delta t_n)\Big]^{1-\delta_{\kappa_n,k}}, \tag{6}$$

where f_k and F_k are the uncoupled probability and cumulative density functions of pipe $\kappa = k$, respectively, and δ is the Kronecker delta. We utilise these likelihood functions to model the probability density of any coupling configuration of pipes.

S2.2 Bayes factor

To evaluate whether a pair of adjacent trails are coupled, we calculate the Bayes factor of the coupled model M_c and the uncoupled model M_u. The Bayes factor B_{cu} of two models M_c and M_u is given by the ratio of probabilities of observing the data $\mathbf{t}$ given each model, i.e,

$$B_{cu} = \frac{\mathbb{P}(\mathbf{t} \,|\, M_c)}{\mathbb{P}(\mathbf{t} \,|\, M_u)}. \tag{7}$$

For example, if $B_{cu} > 1$ then M_c is preferred over M_u. Here, M_c is the coupled model and M_u is the uncoupled model. Kass & Raftery (1995) state that Bayes factor magnitudes between 10-100 are 'strong' and above 100 are 'decisive'. We define a new parameter $\phi \in \{0, 1\}$ such that $\phi = 1$ indicates the coupled model M_c and $\phi = 0$ indicates the uncoupled model M_u. The Bayes factor can be rewritten in terms of ϕ as

$$B_{cu} = \frac{\mathbb{P}(\mathbf{t} \,|\, \phi = 1)}{\mathbb{P}(\mathbf{t} \,|\, \phi = 0)}. \tag{8}$$

In this form, the Bayes factor can be calculated with MCMC methods. We assume a prior distribution for $\phi \sim \text{Bernoulli}(\frac{1}{2})$. Using the likelihood functions of the coupled and uncoupled models, the posterior distribution $\mathbb{P}(\mathbf{t} \mid \phi)$ can be sampled (using e.g., the Metropolis-Hastings algorithm) from which the Bayes factor can be calculated.

S3. INVERSION TESTS

Inversions are performed on synthetic data to test the accuracy and sensitivity of our inversion method. Here, we apply uniform prior distributions to assess the effectiveness of the likelihood function alone. We nondimensionalise the parameters $(\Gamma, \overline{\sigma_T}, s_T)$ to $\Gamma^* = \Gamma/\overline{\sigma_T}$ and $s_T^* = s_T/\overline{\sigma_T}$.

S3.1 One pipe

Inversions of a single pipe trail perform well if the true $s_T^* < 1$. When $s_T^* > 1$, the distribution of Δt begins to be significantly truncated for $\Delta t < 0$ and tends towards a uniform distribution for larger s_T^*. Henceforth, we analyse simulations with $s_T^* < 1$.

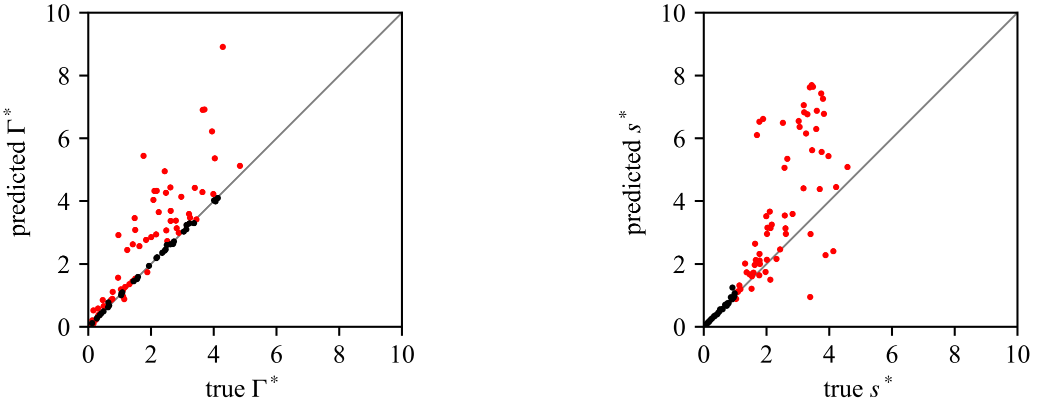

Figure S2: Showing inversion results for simulated synthetic data. Each simulation uses a pair of values for Γ^* and s_T^* to generate a sequence of 1000 venting times. (a) Predicted mean Γ^* versus the true assigned Γ^* for that simulation. (b) Predicted mean s_T^* versus the true assigned s_T^* for that simulation. Black points represent inversion results from simulations with true $s^* < 1$ and red points with true $s^* > 1$.

S3.2 Two pipes

We similarly perform inversions on synthetic data from simulations of two uncoupled pipes, shown in Fig. S3, and two coupled pipes, shown in Fig. S4. In these figures, each point represents results from Bayesian inversion applied to a simulated sequence of 40 venting times from two pipes. The number of venting times was chosen to investigate the level of uncertainty in the inversion for a pair of Levant pipe trails, which each typically comprise ~20 venting times. In each case, the predicted Γ^* is in agreement with the true value; the uncertainty in the inference of s^* increases with increasing true s^*.

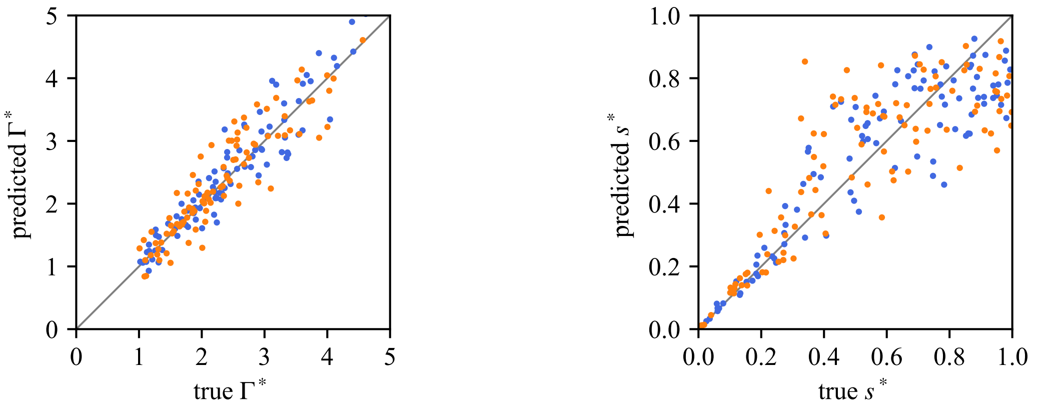

Figure S3: Showing inversion results for simulated synthetic data of two uncoupled pipes. Each simulation uses a pair of values for Γ^* and s^*_T to generate 40 events in total. The inversion of each simulation generates two points, one for pipe 1 (blue) and one for pipe 2 (orange). (a) Predicted mean Γ^* versus the true assigned Γ^* for that simulation. (b) Predicted mean s^*_T versus the true assigned s^*_T for that simulation.

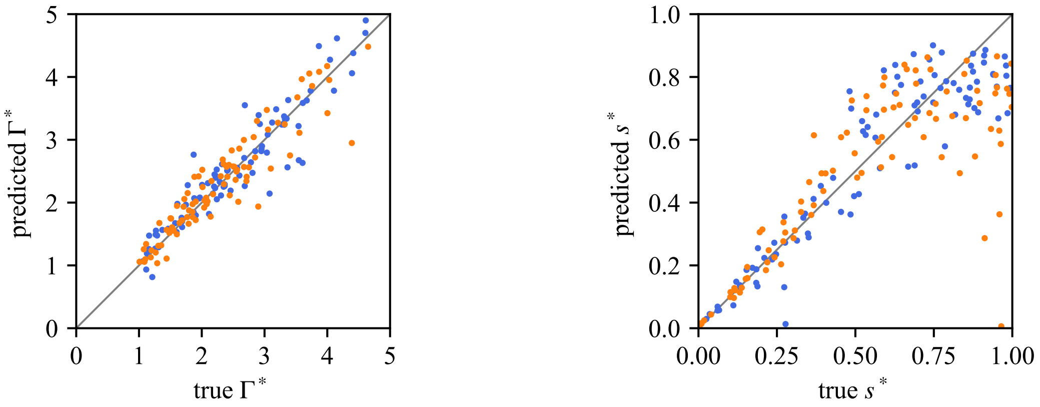

Figure S4: Showing inversion results for simulated synthetic data of two coupled pipes. Each simulation uses a pair of values for Γ^* and s^*_T to generate 40 events in total. The inversion of each simulation generates two points, one for pipe 1 (blue) and one for pipe 2 (orange). (a) Predicted mean Γ^* versus the true assigned Γ^* for that simulation. (b) Predicted mean s^*_T versus the true assigned s^*_T for that simulation.

S4. OCEANUS POSTERIOR DISTRIBUTIONS

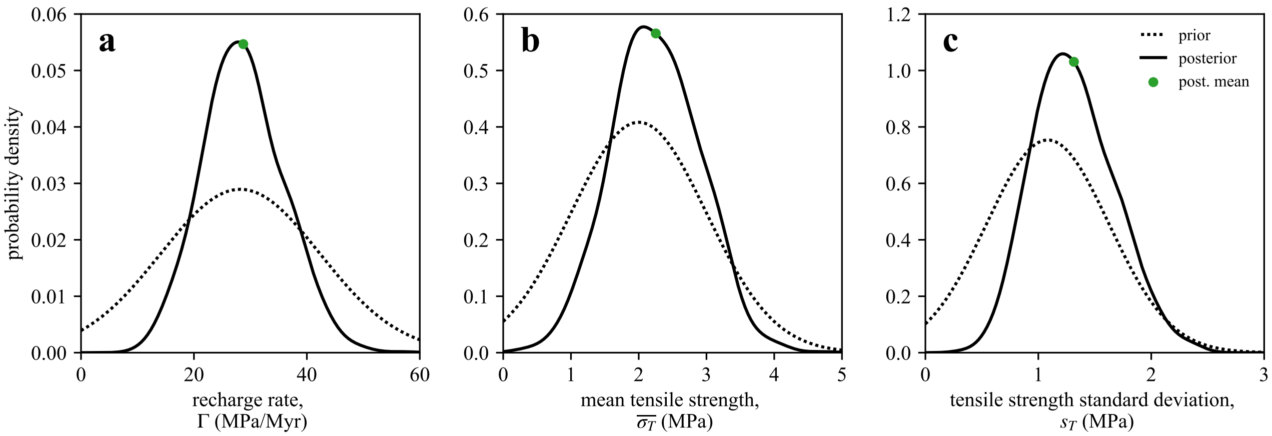

Figure S5: Results of Bayesian inference applied to Oceanus trail. (a) Prior and posterior distributions of the recharge rate, Γ, with posterior mean 28 MPa/Myr. (b) Prior and posterior distributions of the mean tensile strength, $\overline{\sigma_T}$ with posterior mean 2.3 MPa. (c) Prior and posterior distributions of the tensile strength standard deviation, s_T with posterior mean 1.3 MPa.

S5. PRESSURE COUPLING

Whether pipe trails are coupled or not leads to profound changes in the resulting pattern of pockmarks. Fig. S6 demonstrates this clearly with a pair of pipes in each scenario. The uncoupled pipes have independent pressure histories hence the pattern of pockmarks leads to an alternation between each pipe venting. In contrast, for a system of two coupled pipes, after either pipe vents both pressures reset to $\Delta p = 0$ and sample new tensile strengths. Therefore, if one pipe vents, the other is temporarily inhibited from venting. This leads to a complementary pockmark series, where the periods of quiescence of one pipe correspond to activity in the second pipe. In comparison to the field data (Fig. 1d), uncoupled behaviour is exhibited by pipe trails 10, 11 and 12, while coupled behaviour is most pronounced in trails 3, 4 and 5.

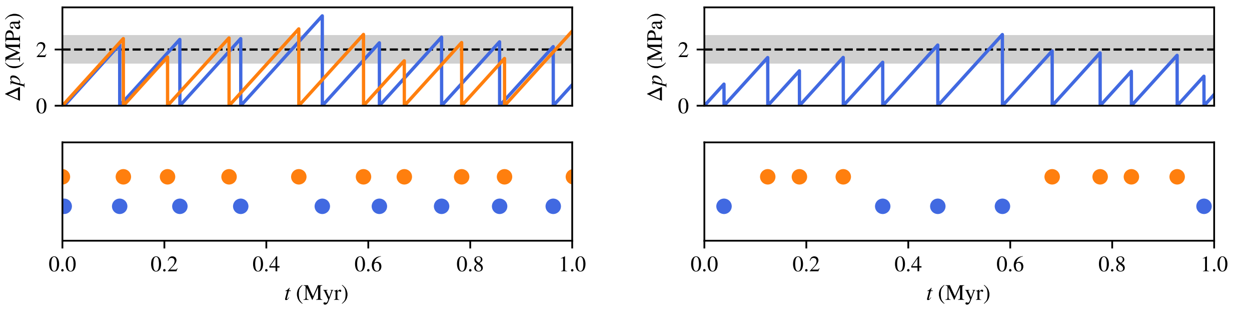

Figure S6: Stochastic realisations of two pipes, each with $\Gamma = 20$ MPa/Myr, $s = 0.5$ MPa and $\overline{\sigma_T} = 2$ MPa. Top plots show pressure evolution, where $\Delta p = p - \sigma_{\min}$ and bottom plots show the corresponding pockmark patterns. Left: uncoupled, right: coupled. Dashed horizontal lines indicate the mean tensile strength $\overline{\sigma_T}$; grey bars contain tensile strengths within one standard deviation of the mean ($\overline{\sigma_T} \pm s_T$).

S6. TRIPLE-WISE INFERENCE

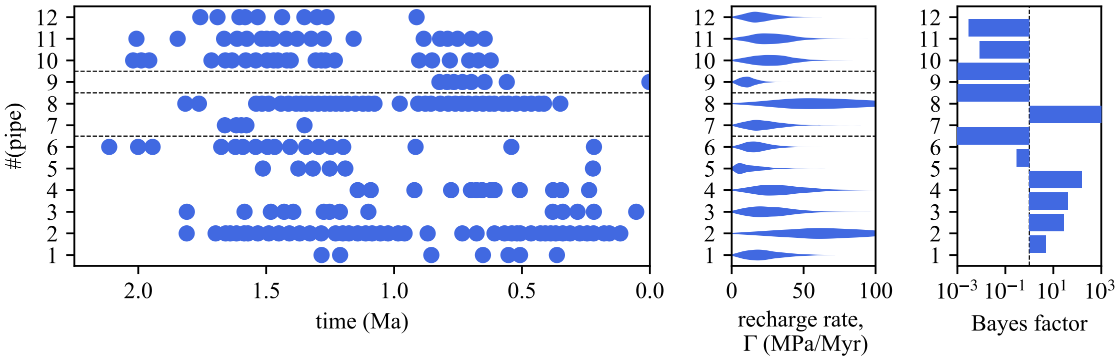

Figure S7: Results of triple-wise Bayesian inference applied to Levant margin data. (a) Time-transformed data from Oppo et al. (2021). Dashed lines divide pipe clusters that are separated by more than 10 km. (b) Violin plot of posterior recharge rate distributions for each pipe trail. (c) Bayes factors of pairwise pipe analysis, where a positive value implies the coupled model is more likely.

S7. CANDIDATE OVERPRESSURE MECHANISMS

S7.1 Tectonic compression

Parameter	Description	mean	std. dev.	min.	max.	Reference
$\Delta P/\Delta e_{xx}$ (MPa)	overpressure per % strain	1.2	0.2	0.5	2.0	Obradors-Prats et al. (2016)
Δe_{xx}	horizontal strain	5	2	1	10	
Δt_e (Myr)	strain duration	5.5	0.5	5	6	Cartwright et al. (2021)

The model for tectonic compression developed by Kearney et al. (2023) is highly simplified; to ensure an accurate estimation of Γ_s we use results from previous numerical modelling of tectonic compression (Obradors-Prats et al. 2016). **?** estimate overpressures between 11–14.2 MPa from 10% strain at different rates, implying that 1.1–1.4 MPa of overpressure is generated per % strain. Using seismic imaging, we estimate the strain at Oceanus to range from 1% to 10%, which we assume has been accumulating since the Messinian Salinity Crisis, between 5 Ma to 6 Ma.

S7.2 Pressure diffusion

Parameter	Description	mean	std. dev.	min.	max.	Reference
h_s (m)	sandstone thickness	150	50	50	200	Cartwright et al. (2021)
h_m (m)	mudstone thickness	2500	250	2000	3000	
α_s	sandstone Biot coefficient	0.62	0.17	0.38	0.83	Ge & Garven (1992)
α_m	mudstone Biot coefficient	0.68	0.35	0.30	0.98	
v_s	sandstone Poisson ratio	0.24	0.04	0.20	0.30	
v_m	mudstone Poisson ratio	0.25	0.05	0.15	0.30	
$\log_{10} K_s$ ($\log_{10}$ GPa)	sandstone bulk modulus	9.5	0.5	9	11	Chang et al. (2013)
$\log_{10} K_m$ ($\log_{10}$ GPa)	mudstone bulk modulus	9.5	0.75	8	11	
ϕ_s	sandstone porosity	0.22	0.01	0.19	0.24	Ortega et al. (2018)
ϕ_m	mudstone porosity	0.20	0.05	0.05	0.30	Yang & Aplin (2007)
$\log_{10} k_m$ ($\log_{10}$ m^2)	(log) mudstone permeability	-19	0.5	-22	-18	
η (mPa s)	water viscosity	0.3	0.1	0.1	0.5	Abramson (2007)
c_ℓ (10^{-11} Pa^{-1})	water compressibility	4.0	0.1	3.7	4.3	Fine & Millero (1973)

S7.3 Flow focusing: marginal uplift

Parameter	Description	mean	std. dev.	min.	max.	Reference
θ (°)	tilt angle	3	1	0	5	Cartwright et al. (2021)
ρ_m (kg/m^3)	mudstone density	2350	100	2200	2600	
ρ_ℓ (kg/m^3)	water density	1060	100	1000	1200	
L_s (m)	sandstone length	5000	1000	500	10000	
Δt_u (Myr)	uplift duration	2	0.5	1	3	Oppo et al. (2021)

Flow focusing due to marginal uplift can lead to overpressure generation. For a flat sandstone of length L_s in a mudstone with pressure gradient $\rho_m g$, uplifting one side by dz leads to an equilibration of pressures at the new sandstone centroid, d$z/2$ (Flemings et al. 2002). Therefore, tilting the sandstone by an angle θ gives an overpressure of

$$\Delta p = \tfrac{1}{2}(\rho_m - \rho_\ell) g L_s \sin\theta, \tag{9}$$

at the top of the reservoir. The corresponding overpressure rate is

$$\frac{\partial p}{\partial t} = \tfrac{1}{2}(\rho_m - \rho_\ell) g L_s \dot{\theta} \cos\dot{\theta} t, \tag{10}$$

where $\dot{\theta}$ is the angular tilting rate. For simplicity of interpretation, we take the time-average of this overpressure rate, given by

$$\Gamma = \frac{(\rho_m - \rho_\ell) g L_s \sin\theta}{2\Delta t_u}. \tag{11}$$

S7.4 Flow focusing: folding

Parameter	Description	mean	std. dev.	min.	max.	Reference
ρ_m (kg/m^3)	mudstone density	2350	100	2200	2600	Cartwright et al. (2021)
ρ_ℓ (kg/m^3)	water density	1060	100	1000	1200	
h_s (m)	sandstone thickness	150	50	50	200	
Δt_f (Myr)	folding duration	5.5	0.5	5	6	

If the sandstone reservoir has a growing parabolic profile, then the overpressure rate generated at the crest by flow focusing is given by (Flemings et al. 2002)

$$\Gamma = \frac{2(\rho_m - \rho_\ell) g \Delta h_f}{3\Delta t_f} \tag{12}$$

where the factor of $2/3$ appears because the sandstone and mudstone pressures equilibrate at $\Delta h/3$.

S7.5 Disequilibrium compaction

Parameter	Description	mean	std. dev.	min.	max.	Reference
ρ_{ps} (kg/m^3)	post-salt sediment density	2000	100	1800	2500	Cartwright et al. (2021)
h_{ps} (m)	post-salt sediment thickness	355	25	300	400	
Δt_c (Myr)	duration	5	0.5	4	6	

Disequilibrium compaction due to post-salt sedimentation contributes to overpressure in the North Levant Basin. For a change in post-salt sediment thickness Δh_{ps} over a time Δt_c with density ρ_{ps}, the maximum overpressure rate is given by the change in total stress,

$$\Gamma = \frac{\rho_{ps} g \Delta h_{ps}}{\Delta t_c} \tag{13}$$

References

Abramson, E. H. (2007), ‘Viscosity of water measured to pressures of 6 GPa and temperatures of 300°C’, *Physical Review E* **76**(5), 051203.

Cartwright, J., Kirkham, C., Foschi, M., Hodgson, N., Rodriguez, K. & James, D. (2021), ‘Quantitative reconstruction of pore-pressure history in sedimentary basins using fluid escape pipes’, *Geology* **49**(5), 576–580.

Chang, K. W., Hesse, M. A. & Nicot, J.-P. (2013), ‘Reduction of lateral pressure propagation due to dissipation into ambient mudrocks during geological carbon dioxide storage’, *Water Resources Research* **49**(5), 2573–2588.

Fine, R. A. & Millero, F. J. (1973), ‘Compressibility of water as a function of temperature and pressure’, *The Journal of Chemical Physics* **59**(10), 5529–5536.

Flemings, P. B., Stump, B. B., Finkbeiner, T. & Zoback, M. (2002), ‘Flow focusing in overpressured sandstones: Theory, observations, and applications’, *American Journal of Science* **302**(10), 827–855.

Ge, S. & Garven, G. (1992), ‘Hydromechanical modeling of tectonically driven groundwater flow with application to the Arkoma foreland basin’, *Journal of Geophysical Research: Solid Earth* **97**(B6), 9119–9144.

Kass, R. E. & Raftery, A. E. (1995), ‘Bayes factors’, *Journal of the American Statistical Association* **90**(430), 773–795.

Kearney, L. M., MacMinn, C. W., Katz, R. F., Kirkham, C. & Cartwright, J. (2023), ‘Episodic, compression-driven fluid venting in layered sedimentary basins’, *Proceedings of the Royal Society A: Mathematical, Physical and Engineering Sciences* **479**(2274), 20220654. (doi:10.1098/rspa.2022.0654).

Obradors-Prats, J., Rouainia, M., Aplin, A. C. & Crook, A. J. (2016), ‘Stress and pore pressure histories in complex tectonic settings predicted with coupled geomechanical-fluid flow models’, *Marine and Petroleum Geology* **76**, 464–477.

Oppo, D., Evans, S., Iacopini, D., Kabir, S. M., Maselli, V. & Jackson, C. A.-L. (2021), ‘Leaky salt: Pipe trails record the history of cross-evaporite fluid escape in the northern levant basin, eastern mediterranean’, *Basin Research* **33**(3), 1798–1819.

Ortega, J., Hebert, R. & Gellman, Y. (2018), The dynamic Tamar reservoir – insights from five years of production, *in* ‘Scientific Conference: Eastern Mediterranean–an emerging major petroleum province’, pp. 48–49.

Yang, Y. & Aplin, A. C. (2007), ‘Permeability and petrophysical properties of 30 natural mudstones’, *Journal of Geophysical Research: Solid Earth* **112**(B3).
